# Effects of detraining and retraining on muscle energy-sensing network and meteorin-like levels in obese mice

**DOI:** 10.1186/s12944-018-0751-3

**Published:** 2018-04-27

**Authors:** Ju Yong Bae, Jinhee Woo, Sunghwun Kang, Ki Ok Shin

**Affiliations:** 1 Laboratory of Exercise Biochemistry, Department of Physical Education, College of Arts and Physical Education, Dong-A University, 37 Nakdong-daero 550 beon-gil, Hadan-dong, Saha-gu, Busan, 604-714 Republic of Korea; 2 Laboratory of Exercise Physiology, Division of Sport Science, Kangwon National University, 1 Kangwondaehak-gil, Chuncheon-si, Gangwon-do 24341 Republic of Korea

**Keywords:** Detraining, Retraining, AMPK, SIRT-1, PGC-1α, Meteorin-like

## Abstract

**Background:**

Increased intramuscular peroxisome proliferator-activated receptor gamma coactivator-1α (PGC-1α) with exercise directly or indirectly affects other tissues, but the effector pathway of PGC-1α has not been clearly elucidated. The purpose of this study was to investigate the effect of exercise and/or dietary change on the protein levels of the soleus muscle energy-sensing network and meteorin-like (Metrnl), and additionally to analyze the detraining and retraining effects in high-fat diet (HFD)-induced obese mice.

**Methods:**

One hundred male C57BL/6 mice were divided into normal-diet + sedentary (CO, *n* = 20) and HFD + sedentary (HF, *n* = 80) groups, and obesity was induced in the HF group through consumption of a 45% HFD for 6 weeks. The HF group was subdivided into HF only (*n* = 20), HF + training (HFT, *n* = 20), dietary change + sedentary (HFND, *n* = 20), and HFND + training (HFNDT, *n* = 20) groups, and the mice in the training groups underwent a treadmill training for 8 weeks, 5 times per week, 40 min per day. The HFT and HFNDT groups underwent 8-week training, 8-week detraining, and 4-week retraining.

**Results:**

An 8-week training was effective in increasing the protein levels of soleus muscle AMP-activated protein kinase (AMPK), PGC-1α, and plasma Metrnl in the obese mice (*P* < 0.05). Moreover, exercise in obesity reduced body weight (*P* < 0.05), and exercise with dietary conversion was effective in reducing body weight (*P* < 0.05) and fat mass (*P* < 0.05) after 8-week training. 8-week detraining restored the increased protein level to the pre-exercise state, but, the previous exercise effect in body weight and fat mass (*P* < 0.05) of the HFNDT group remained until the end of 4-week detraining. 4-week retraining was effective in increasing the protein levels of soleus muscle AMPK, PGC-1α, blood Metrnl (*P* < 0.05), and reducing in body weight (*P* < 0.05) and fat mass (*P* < 0.05), when retraining with dietary change.

**Conclusions:**

The results of this study suggest that regular exercise is indispensable to reduce body weight and fat mass through upregulation of the muscle energy-sensing network and Metrnl protein levels, and retraining with dietary change is necessary to obtain the retraining effects more quickly.

**Electronic supplementary material:**

The online version of this article (10.1186/s12944-018-0751-3) contains supplementary material, which is available to authorized users.

## Background

A chronic high fat diet (HFD) inhibits the use of fat as an energy source and increases the risk of resistance to satiety, and can alter the nervous system activity and metabolic processes associated with energy storage and consumption [1]. An imbalance of energy metabolism due to HFD induces adult diseases, such as metabolic syndrome, cardiovascular disease, and type 2 diabetes [2].

Obese patients with hypertension, metabolic disorders, and kidney disease may require medication and surgical intervention to reduce weight and body fat [3]. However, except for chronically obese patients with complications, exercise is one of the most effective ways to prevent and treat obesity [4].

Exercise promotes muscle energy-sensing network proteins, including AMP-activated protein kinase (AMPK), Sirtuin-1 (SIRT-1), and peroxisome proliferator-activated receptor gamma coactivator-1α (PGC-1α), through repeated muscle contraction and relaxation [5]. Among the muscle energy-sensing network proteins, PGC-1α is a major regulator that strongly induces mitochondrial biogenesis, resulting in the conversion of fatty acids into energy sources in the mitochondria [6], and intramuscular PGC-1α is increased through various physical activities, including both regular aerobic exercise and resistance exercise. [7]. Increased intramuscular PGC-1α with exercise directly or indirectly affects other tissues, but the effector pathway of PGC-1α has not been clearly elucidated, since increased intramuscular PGC-1α does not release from muscles.

Meteorin-like protein (Metrnl), also known as Subfatin, has a molecular weight of 35 kD, and is transcribed similarly to Meteorin, which is mainly expressed in the brain [8]. A recent study has reported that upregulated PGC-1α after exercise increased Metrnl in muscle tissue, and Metrnl then transfers the positive effects of PGC-1α to other tissues. Increased muscle-induced Metrnl is released into the blood and, through circulation, affects other tissues, such as the liver and adipose tissue [9].

Exercise improves the ability to transport fatty acids into the skeletal muscle [10], promotes mitochondrial biogenesis [11], and plays a role in reducing weight and fat mass. However, despite the positive effects of exercise, people often stop exercising for a variety of reasons, thereby incurring detraining effects. Although the effect of detraining is likely to be worse with continuous HFDs, few studies on detraining and retraining are currently available.

Therefore, the purpose of this study was to analyze the effects of regular treadmill exercise and dietary change on the protein levels of the muscle energy-sensing network and of Metrnl in HFD-induced obese mice. Additionally, we analyzed detraining and retraining effects after regular training and dietary change.

## Methods

### Animals

One hundred 4-week-old male C57BL/6 mice were obtained from Samtako (Osan, Korea), and acclimatized for a week under standardized conditions in an animal facility (Dong-A University College of Medicine Animal Laboratory). The animal experiments gained approval from the Dong-A University Medical School Institutional Animal Care and Use Committee and all procedures were conducted in accordance with the Committee’s Guidelines on Animal Experiments recommendations.

### Experimental design

#### Period of obesity induction

After 1 week of environmental adaptation, all animals were randomly assigned to a normal diet + sedentary group (CO, *n* = 20) or an HFD + sedentary group (HF, *n* = 80). For 6 weeks, the HF group was fed a 45% fat diet (45% lipid, 35% carbohydrate, and 20% protein) to induce obesity, whereas the CO was fed a standard diet. All animals had free access to tap water, and dietary intakes were recorded every morning (09:00); body weight was also measured every week at the same time (09:30).

#### Period of treadmill training

After the inducing-obesity period was complete, the HF group was randomly divided into an HF group (*n* = 20), an HF + training group (HFT, *n* = 20), a normal diet + sedentary group (HFND; dietary change, *n* = 20), and an HFND + training group (HFNDT, *n* = 20). During the 8-week treadmill exercise period, the mice were treated with diets and exercises corresponding to each group, and five mice from each group were dissected 48 h after the end of the last exercise.

#### Period of treadmill detraining

To study the effects of detraining after the 8-week training, treatment of the HF groups was changed to detraining for the two training groups: HF only (*n* = 15) no change, detraining after HFT treatment (HFT-DT, *n* = 15); HFND (*n* = 15) no change, detraining after HFNDT treatment (HFNDT-DT, *n* = 15). During the period of 8-week detraining, the HFT-DT and HFNDT-DT groups stopped exercise, and five mice from each group were dissected after the 4- and 8-week treatments.

#### Period of treadmill retraining

To study the effects of retraining after the 8-week detraining, the treatment of HF groups was changed to retraining for the two detrained groups: HF only (*n* = 5) no change, retraining after HFT-DT treatment (HFT-RT, *n* = 5); HFND (*n* = 5) no change, retraining after HFNDT treatment (HFNDT-RT, *n* = 5). After the period of 8-week detraining, the HFT-RT and HFNDT-RT groups underwent treadmill training for 4 weeks, and five mice from each group were dissected 48 h after the end of the last exercise.

### Treadmill training and retraining

The training protocol which did not induce muscle damage used in a previous study was applied in this study [12]. Exercise intensity consisted of 5 m/minute for five minutes, 12 m/minute for five minutes and 18 m/minute for 20 min at 0% slope for the first 4 weeks of training session (low intensity). For the last 4 weeks, exercise intensity was increased to 10 m/minute for five minutes, 16 m/minute for five minutes, and 22 m/minute for 30 min at 0% slope (moderate intensity).

Retraining intensity was conducted at the same intensity as treadmill during the last 4 weeks. Because of previous exercise experience, the mice did not undergo a period of training adaptation.

### Blood and tissue samplings

To rule out temporary training effects, tissue sampling was conducted 48 h after the completion of the last exercise. After complete anesthesia (ethyl ether), blood samples (1 mL) were obtained from the abdominal vena cava via syringes. Plasma was collected using centrifugation of heparinized blood at 3000 rpm for 15 min. After blood sampling, abdominal visceral fat and right leg muscle tissue were excised and weighed. Lipid profiles were analyzed immediately using plasma (Additional files 1, 2, and 3), and tissues were stored at − 80 °C until analysis.

### Plasma Metrnl protein analysis

Plasma Metrnl protein concentrations were determined through Enzyme-Linked Immunosorbent Assay (ELISA), using a Mouse Meteorin-like/METRNL DuoSet ELISA Kit (Catalog number DY6679, R&D Systems, MN, USA). The procedures were performed according to the manufacturer’s instructions. A total of 100 μL of sample was added per well, and the wells were incubated for 2 h after covering with an adhesive strip. After washing, 100 μL of the Detection Antibody was added and then incubated for 2 h at room temperature. After washing, 100 μL of the Streptavidin-HRP was added to each well and the wells were incubated for 20 min at room temperature in the absence of direct sunlight. After washing, 100 μL of the Substrate Solution was added to each well and the wells were incubated for 20 min at room temperature in the absence of direct sunlight. Thereafter, 50 μL of Stop Solution was add to each well and the optical density was determined immediately, using a microplate reader at 450 nm. The result of concentration was calculated by multiplying sample by dilution factor.

### Soleus muscle protein analysis

Muscle AMPK, SIRT-1, PGC-1α, and Metrnl protein levels were analyzed using Western blotting. To extract protein from the soleus muscle, the tissues were lysed in a 200 μL radioimmunoprecipitation assay buffer. The tissue was homogenized and centrifuged for 30 min at 14,000 rpm. The protein concentration of the supernatant was measured using a BCA protein assay kit (PIERCE, USA). Protein samples were mixed with Laemmli sample buffer and placed in a boiling water bath for five minutes. Samples of equal protein content were resolved using SDS-polyacrylamide gel electrophoresis on a 10 or 12% gel, and transferred to a membrane. The membrane was blocked with 5% skim milk in phosphate-buffered saline, and subsequently incubated at 4 °C overnight with primary antibodies (1:1000 dilution) against AMPK (#2532, Cell Signaling, USA), p-AMPK (#2535, Cell Signaling), SIRT-1 (#2028, Cell Signaling), PGC-1α (ab54481, Abcam, USA), and Metrnl (sc-168,581, Santa Cruz, USA). The membrane was incubated with goat anti-mouse or anti-rabbit IgG conjugated secondary antibody for 1 h at room temperature. The signal was developed with an ECL solution (Amersham Pharmacia Biotech, USA) and visualized with an ImageQuant LAS-4000 system (GE Healthcare, Sweden).

### Statistical analysis

All calculations were performed using the Statistical Package for Social Sciences version 22.0 (SPSS Inc., Chicago, IL, USA) and presented as means ± standard error. The change in body weight induced with HFD was analyzed using independent t-test and ANOVA. One-way ANOVA and Duncan’s post-hoc analysis were performed for any intergroup difference observed. A statistically significant level was set at *p* < 0.05.

## Results

**Fig. 1:**
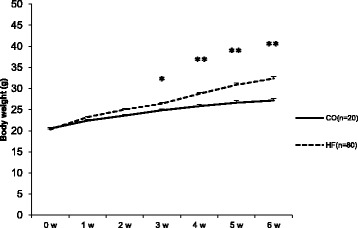
Changes of body weight during periods of HFD. Body weight changes during period of HFD to induce obesity. Values are presented mean ± SE. CO; Normal-diet + sedentary group, HF; High-fat diet + sedentary group. ^*^ *p* < 0.05, ^**^ *p* < 0.01; Significant difference between CO and HF group

To induce obesity, the experimental animals were fed a 45% HFD for 6 weeks before the treadmill training. A significant difference was found between groups after 3 weeks of obesity induction (1.57 g, *p* < 0.05), and the difference in body weight gradually increased until the end of this period (5.18 g, *p* < 0.001) (Fig. 1).

### Treadmill training effects

**Fig. 2:**
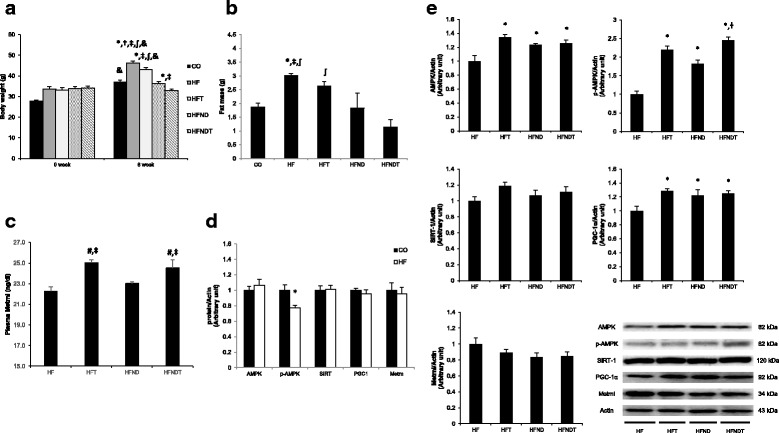
Changes after 8 weeks of training. Changes of body weight (**a**), fat mass (**b**), plasma Metrnl protein levels (**c**), soleus muscle proteins level of CO and HF group (**d**), and soleus muscle proteins level of experimental groups (**e**) after 8 weeks of training are presented. Values are presented mean ± SE. CO; Normal-diet + sedentary group, HF; High-fat diet + sedentary group, HFT; HF + Training group, HFND; a normal diet + sedentary group (dietary change group), HFNDT; HFND + Training group. ^*^ *p* < 0.05; vs CO group, ^†^ *p* < 0.05; vs HFT, ^⧧^ *p* < 0.05; vs HFND, ^∫^*p* < 0.05; vs HFNDT, ^&^ *p* < 0.05; vs before

After 8 weeks of training, the body weights of the HF group were significantly higher than those of all other groups (*p* < 0.05). The body weights of the HFT group were significantly higher than those of the HFND and HFNDT groups (*p* < 0.05). In addition, the body weights of the HFNDT group were significantly lower than those of the HFND group (*p* < 0.05). When comparing body weight changes before and after exercise, the body weights of the CO group had increased after exercise (9.18 g, *p* < 0.05), and those of the HF (12.51 g, *p* < 0.05) and HFT groups (10.04 g, *p* < 0.05) had also increased after exercise (Fig. 2a).

The abdominal fat mass of the HF group was significantly higher than those of the CO, HFND, and HFNDT groups (*p* < 0.05). In addition, the abdominal fat mass of the HFT group was significantly higher than that of the HFNDT group (*p* < 0.05) (Fig. 2b).

After 8 weeks of treadmill training, the phosphor-AMPK protein level of the HF group was significantly lower than that of the CO group (*p* < 0.05). The total AMPK protein levels in the soleus muscles of the HFT, HFND, and HFNDT groups were significantly higher than that in the HF group (*p* < 0.05). The levels of phospho-AMPK protein in the HFT, HFND, and HFNDT groups were significantly higher than that in the HF group (*p* < 0.05), and the protein level in the HFNDT group was significantly higher than that in the HFND group (*p* < 0.05). PGC-1α protein levels in the soleus muscle of the HFT, HFND, and HFNDT groups were significantly higher than that in the HF group (*p* < 0.05). However, SIRT-1 and Metrnl protein levels in the soleus muscle were not significantly different between the groups (Fig. 2d, e).

The plasma Metrnl protein levels in the HFT (25.02 ± 0.27 ng/mL) and HFNDT (24.52 ± 0.80 ng/mL) groups were significantly higher than those in the HF (22.27 ± 0.42 ng/mL) and HFND groups (23.02 ± 0.17 ng/mL) (*p* < 0.05) after 8 weeks of training (Fig. 2c).

### Detraining effects

**Fig. 3:**
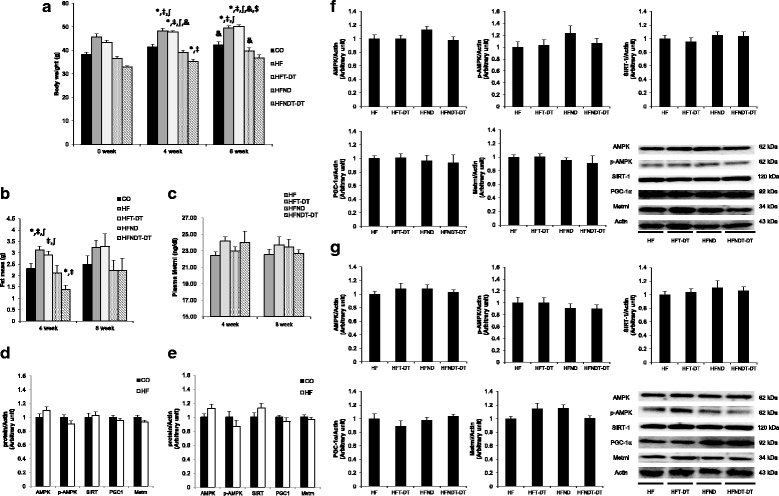
Changes after 4 and 8 weeks of detraining. Changes of body weight (**a**), fat mass (**b**), plasma Metrnl protein levels (**c**), soleus muscle proteins level of CO and HF group after 4-week detraining (**d**), soleus muscle proteins level of CO and HF group after 8-week detraining (**e**), soleus muscle proteins level of experimental groups after 4-week detraining (**f**), and soleus muscle proteins level of experimental groups after 8-week detraining (**g**) are presented. Values are presented mean ± SE. CO; Normal-diet + sedentary group, HF; High-fat diet + sedentary group, HFT-DT; HF + Detraining group, HFND; a normal diet + sedentary group (dietary change group), HFNDT-DT; HFND + Detraining group. ^*^ *p* < 0.05; vs CO group, ^⧧^ *p* < 0.05; vs HFND, ^∫^*p* < 0.05; vs HFNDT, ^&^ *p* < 0.05; vs 0-week, ^$^ *p* < 0.05; vs 4-week

After 4 weeks of detraining, the body weights of the HF and HFT-DT groups were significantly higher than those of the CO and HFND groups (*p* < 0.05), and the body weight of the HFNDT-DT group was significantly lower than those of the HFND group (*p* < 0.05). After 8 weeks of detraining, the body weights of the HF and HFT-DT groups were significantly higher than those of the CO, HFND, and HFNDT-DT groups (*p* < 0.05). When comparing the weight change over time, the body weights of the CO group (4.11 g) and the HFNDT-DT group (3.91 g) significantly increased after 8 weeks of detraining compared to the weights before detraining (*p* < 0.05). The body weights of the HFT-DT group increased after 4 weeks of detraining compared to those before detraining (4.56 g, *p* < 0.05), and after 8 weeks of detraining compared to 4 weeks of detraining (2.3 g, *p* < .05) (Fig. 3a). After 4 weeks of detraining, the abdominal fat mass of the HF group was significantly higher than those of the CO, HFND, and HFNDT-DT groups, and that of the HFT-DT group was significantly higher than those of the HFND and HFNDT-DT groups (*p* < 0.05). Moreover, the abdominal fat mass of the HFNDT-DT group was significantly lower than those of the CO and HFND groups (*p* < 0.05). However, after 8 weeks of detraining, no statistically significant differences were found in abdominal visceral fat mass among the groups (Fig. 3b). The protein levels of total AMPK, phospho-AMPK, SIRT-1, PGC-1, and Metrnl in the soleus muscle were not significantly different among the groups after 4 and 8 weeks of detraining. The plasma Metrnl protein levels of the HF (22.42 ± 0.46 ng/mL) and HFND groups (22.98 ± 0.53 ng/mL) were lower than those of the HFT-DT (24.16 ± 0.52 ng/mL) and HFNDT-DT groups (23.98 ± 1.40 ng/mL) after 4 weeks of detraining, but the difference was not statistically significant. In addition, the plasma Metrnl protein levels of the HFT-DT (from 24.16 ± 0.52 ng/mL to 23.71 ± 0.98 ng/mL) and HFNDT-DT groups (from 23.98 ± 1.40 ng/mL to 22.69 ± 0.44 ng/mL) tended to decrease after 8 weeks of detraining compared to plasma Metrnl protein levels after 4 weeks of detraining (Fig. 3c).

### Retraining effects

**Fig. 4:**
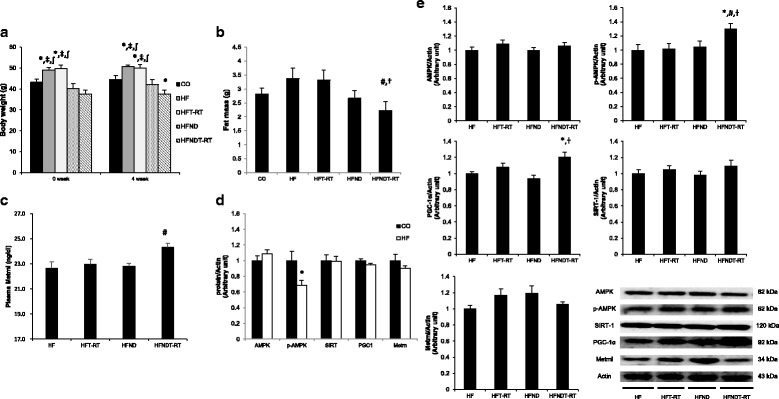
Changes after 4 weeks of retraining. Changes of body weight (**a**), fat mass (**b**), plasma Metrnl protein levels (**c**), soleus muscle proteins level of CO and HF group (**d**), and soleus muscle proteins level of experimental groups (**e**) after 4 weeks of retraining are presented. Values are presented mean ± SE. CO; Normal-diet + sedentary group, HF; High-fat diet + sedentary group, HFT-RT; HF + Retraining group, HFND; a normal diet + sedentary group (dietary change group), HFNDT-RT; HFND + Retraining group. ^*^ *p* < 0.05; vs CO group, ^⧧^ *p* < 0.05; vs HFND, ^∫^*p* < 0.05; vs HFNDT, ^#^ *p* < 0.05; vs HF

After 4 weeks of retraining, the body weights of HF and HFT-RT groups were significantly higher than those of the CO, HFND, and HFNDT-RT groups (*p* < 0.05). The abdominal visceral fat mass of the HFNDT-RT group was significantly lower than those in the HF and HFT-DT groups after 4 weeks of retraining (*p* < 0.05) (Fig. 4a, b). After 4 weeks of treadmill retraining, the phosphor-AMPK protein level in the HF group was significantly lower than that in the CO group (*p* < 0.05). The protein level of phospho-AMPK in the soleus muscle of the HFNDT-RT group was significantly higher than those of the HF, HFT, and HFND groups after 4 weeks of retraining (*p* < 0.05). The protein level of PGC-1α in the HFNDT-RT group was significantly higher than those of the HF and HFND groups (*p* < 0.05) (Fig. 4d, e). The plasma Metrnl protein level in the HFNDT-RT group (24.32 ± 0.32 ng/mL) was significantly higher than that of the HF group (22.66 ± 0.51 ng/mL) (*p* < 0.05) (Fig. 4c).

## Discussion

AMPK is activated in an energy stress state that increases the AMP/ATP ratio, resulting in a promotion of ATP production through AMPK phosphorylation [13]. In addition, AMPK is highly expressed in brain and brown adipose tissue, and participates in the energy metabolism of various tissues [14]. However, in cases of obesity, the muscle AMPK activity was inhibited and the mitochondrial activity was reduced [15, 16].

In this respect, the importance of AMPK activity to reduce obesity-related diseases, such as diabetes, metabolic syndrome, and cardiovascular disease was emphasized, and the development of drugs related to obesity and metabolic syndrome using quercetin, genistein, and resveratrol, which act as AMPK promoters, is actively under way [17]. Increased AMPK promotes SIRT-1 action through increasing the NAD+/NADH ratio, and increased AMPK-induced SIRT-1 induces mitochondrial biogenesis through deacetylating PGC-1α [18]. PGC-1α is a major regulator of mitochondrial biogenesis and induces a variety of protein expressions through increasing gene transcription factors, such as PPARα and ERRα [19].

The muscle energy obesity-related sensing network signaling pathway is an important link in maintaining energy metabolism homeostasis in the body and is used in preventive and therapeutic strategies against type 2 diabetes and metabolic disorders [5]. The results of this study showed that 8-week treadmill training increased AMPK, phospho-AMPK, and PGC-1α protein levels. In a study by Oliveira et al. [20], AMPK decreased as aging progressed, and SIRT-1 and PGC-1α protein levels were increased with AMPK phosphorylation through regular aerobic exercise. A previous study reported that 20-week HFDs decreased AMPK activity and PGC-1α mRNA expression, but both sustained and temporary exercise contributed to AMPK and PGC-1α activity [21], which is consistent with the results of this study. However, in this study, PGC-1α protein level was increased without increasing SIRT-1, suggesting that PGC-1α upregulation was more affected with AMPK phosphorylation [22] rather than the action of SIRT-1 as a deacetylase [23].

One recent study has reported that repetitive contraction of skeletal muscles through exercise induces an increase in PGC-1α protein levels at the final stage of the muscle energy-sensing network, which increases the level of Metrnl protein in muscle tissue, thereby potentially suggesting the possibility of treating metabolic syndrome and inflammatory diseases [9]. In this study, although an increase of Metrnl in the soleus muscle was not observed, we confirmed an increase of Metrnl protein levels in the blood. In a previous study, the Metrnl mRNA level was increased in vastus lateralis after one-time combined exercise, which was somewhat different from this study. These differences may be attributed to the diverse types of exercise involved and the different muscles analyzed, and later studies may be necessary to analyze the levels of Metrnl protein in various muscle and adipose tissues that affect the increase of blood Metrnl protein level.

Regular exercise is effective in weight management through affecting appetite and inflammatory proteins and related hormones [24, 25]. However, detraining effects due to exercise interruption act negatively on the body, and detraining while obese and with continuous HFDs seems to be more negative. Therefore, further studies are needed to prevent the occurrence of detraining effects.

Detraining is defined as the loss of physiological and behavioral exercise-induced adaptation [26]. Detraining results in a decrease in fatty acid oxidation capacity in muscle, liver, and adipose tissue [27], and increases body weight and fat mass [28, 29]. In addition, detraining reduces muscle capillary blood flow through reducing muscle function [27], and negatively affects intramuscular energy metabolism.

The effects of detraining are very diverse and can vary depending on the type of previous exercise undertaken, for example whether resistant, aerobic, or combined exercises. It may also depend on the individual or subject being studied, the duration of the exercise and the duration of the stopped exercise. In this study, the positive effects of exercise on body weight and fat mass had all disappeared after 4 weeks of detraining in the HFT-DT group, but the positive effects lasted longer in HFNDT-DT group.

A previous study reported that 10 weeks of aerobic exercise resulted in an increase in capillary formation of the soleus and plantarum, but that exercise-induced VEGF protein levels and changes in skeletal capillary blood vessels disappeared at only 7 days after stopping exercise [30]. In addition, although 4 weeks of swimming exercise resulted in a positive change in body weight, stable oxygen uptake, serum leptin level, and isoproterenol and citrate synthase activity in adipose tissue, 2 weeks of detraining resulted in negative change in fat metabolism.

In the present study, the 4-week detraining returned exercise-enhanced AMPK, PGC-1α, and plasma Metrnl protein to their before-exercise levels. In a previous study, the exercise-induced effects had disappeared after 1- or 2-week detraining, therefore further studies should be carried out that classify exercise interruption into several stages to obtain definite conclusions about detraining effects.

Previous exercise effects can persist for extended periods of inactivity, and retraining effects are facilitated due to previous exercise experiences [31]. In other words, through a phenomenon known as ‘Muscle memory’, it appears that a person with previous exercise experience can achieve a faster exercise effect through retraining than those without exercise experience [32].

Previous studies related to retraining have been principally human studies, and due to limitations imposed on studies involving humans, most studies have focused on changes of body composition and muscle strength [33–35]. Therefore, more research is needed on protein and hormone changes through regular exercise, and analysis conducted in relation to varying physical conditions, such as involving nutritional excess or deficiency, is also necessary.

In this study, based on previous exercise experience, the mice underwent retraining for 4 weeks. The results of this study demonstrated that 4 weeks of retraining with a dietary change to a normal diet led to a decrease in body weight and abdominal visceral fat, and an increase in the muscle energy-sensing network and in Metrnl protein levels. However, retraining with a continuous HFD was not effective. Therefore, low-calorie dietary change with retraining is necessary to induce the positive effects, and long-lasting exercise intervention may be required in the absence of dietary change. In previous studies comparing body composition between athletic and non-athletic children, no difference in BMI was found between groups, but lean body mass and fat mass differed [36]. In this study, we compared only body weight and abdominal fat mass between groups and could not confirm the difference. Therefore, it is considered that measurement of other body composition parameters would be necessary in future studies to confirm the effects of exercise. Above all, regular exercise is necessary to prevent detraining effects.

This study has some limitations. First, the small sample size is a definite limitation. We used a total of 100 mice, but actually only 5 mice of each group were assigned. However, despite the small sample size, it is meaningful to confirm the effect of training, detraining, and the retraining for 26 weeks continuously. We suggest that a sufficient number of mice are required after sample power testing in subsequent studies. Another limitation of this study is that it was difficult to compare the effects in a time-dependent manner, because this study was a long-term study (over 6 months). In other words, due to the generation gap of mice at the start (4 weeks old) and at the end (32 weeks old) of experiment, comparative analysis of successive effects is limited. Therefore, only intergroup comparison was possible after each intervention.

## Conclusions

To conclude, 8-week training was effective in increasing the protein levels of soleus muscle AMPK, PGC-1α, and blood Metrnl levels in the obese mice. However, 4-week detraining returned the increased protein level to its pre-exercise state, and 4-week retraining was effective in increasing the protein levels of soleus muscle AMPK, PGC-1α, and plasma Metrnl levels when retraining was associated with dietary change. Therefore, the results of this study suggest that regular exercise is indispensable to reduce body weight and fat mass through upregulation of the muscle energy-sensing network and Metrnl protein levels, and retraining with dietary change is necessary to obtain retraining effects more quickly.

## Additional files

Additional file 1: **Table S1.** Lipid profiles and glucose after 8 weeks of training. (DOCX 16 kb)

Additional file 2: **Table S2.** Lipid profiles and glucose after 4 and 8 weeks of detraining. (DOCX 18 kb)

Additional file 3: **Table S3.** Lipid profiles and glucose after 8 weeks of retraining. (DOCX 17 kb)

## Abbreviations

AMPK: AMP-activated protein kinase
HFD: High-fat diet
Metrnl: Meteorin-like
PGC-1α: Peroxisome proliferator-activated receptor gamma coactivator-1α
SIRT-1: Sirtuin-1
