# Supplementary material for: Effects of detraining and retraining on muscle energy-sensing network and meteorin-like levels in obese mice
Source: Lipids Health Dis. 2018 Apr 27;17:97. doi: 10.1186/s12944-018-0751-3 (PMC5924483; doi:10.1186/s12944-018-0751-3)
Supplement: Supplementary file 1 — Table S1. Lipid profiles and glucose after 8 weeks of training. (DOCX 16 kb) [file 12944_2018_751_MOESM1_ESM.docx]

**Table S1** Lipid profiles and glucose after 8 weeks of training

|  | CO | HF | HFT | HFND | HFNDT |
| --- | --- | --- | --- | --- | --- |
| TC (mg/DL) | 84.27 ± 2.09 | 157.75 ± 10.53 ^*,ⵜ,⧧,∫^ | 120.96 ± 6.92 ^*^ | 99.34 ± 4.13 | 89.03 ± 5.58 |
| TG (mg/DL) | 87.41 ± 5.46 | 102.35 ± 5.46 ^∫^ | 95.46 ± 3.26 ^∫^ | 103.14 ± 10.13 | 66.46 ± 6.79 |
| HDL-C (mg/DL) | 44.38 ± 3.71 | 51.38 ± 3.38 | 54.23 ± 1.02 | 45.36 ± 2.44 | 41.21 ± 2.05 |
| LDL-C (mg/DL) | 22.41 ± 3.05 | 85.90 ± 8.21 ^*,ⵜ,⧧,∫^ | 47.63 ± 6.39 | 33.36 ± 4.11 | 34.54 ± 5.36 |
| Glucose (mg/DL) | 225.8 ± 23.82 | 266.0 ± 16.49 ^∫^ | 253.6 ± 33.26 ^∫^ | 185.6 ± 23.12 | 167.2 ± 28.59 |

Values are means±SE, *p<.05; vs CO, ⵜp<.05; vs HFT, ⧧p<.05; vs HFND, ∫p<.05; vs HFNDT, TC; Total Cholesterol, TG; Triglyceride, HDL-C; High Density Lipoprotein-Cholesterol, LDL-C; Low Density Lipoprotein- Cholesterol, CO; Normal diet group (n=5), HF; High fat diet group (n=5), HFT; High fat diet + Training group (n=5), HFND; Dietary change group (n=5), HFNDT; Dietary change + Training group (n=5).

Lipid profiles and glucose analysis

Plasma total cholesterol (TC) and triglyceride (TG) levels were analyzed with commercial TC and TG kits (Asan Pharmaceutical, Korea). High density lipoprotein cholesterol (HDL-c) level was analyzed with HDL-c kits (Shinyang Diagnostics, Korea) and Low density lipoprotein cholesterol (LDL-c) was calculated with the following equation: LDL-c = TC - (HDL-c + TG/5). Blood glucose level was estimated using a GlucoDr glucometer (Allmedicus, Korea).
