# Supplementary material for: Effects of detraining and retraining on muscle energy-sensing network and meteorin-like levels in obese mice
Source: Lipids Health Dis. 2018 Apr 27;17:97. doi: 10.1186/s12944-018-0751-3 (PMC5924483; doi:10.1186/s12944-018-0751-3)
Supplement: Supplementary file 2 — Table S2. Lipid profiles and glucose after 4 and 8 weeks of detraining. (DOCX 18 kb) [file 12944_2018_751_MOESM2_ESM.docx]

**Table S2** Lipid profiles and glucose after 4 and 8 weeks of detraining

|  | | CO | HF | HFT-DT | HFND | HFNDT-DT |
| --- | --- | --- | --- | --- | --- | --- |
| 4  w | TC (mg/DL) | 110.09 ± 9.72 | 208.19 ± 10.84 ^*,⧧,∫^ | 186.79 ± 16.12 ^*,⧧,∫^ | 122.18 ± 8.28 | 106.77 ± 5.61 |
|  | TG (mg/DL) | 91.45 ± 6.13 | 111.21 ± 11.19 | 95.94 ± 11.69 | 112.15 ± 11.95 | 116.77 ± 1.66 |
|  | HDL-C (mg/DL) | 46.66 ± 2.53 | 54.80 ± 1.19 | 60.26 ± 2.39 | 52.20 ± 2.63 | 47.01 ± 5.46 |
|  | LDL-C (mg/DL) | 45.14 ± 8.57 | 131.14 ± 8.87 ^*,⧧,∫^ | 107.35 ± 14.97 ^*,⧧,∫^ | 47.55 ± 4.87 | 36.40 ± 6.15 |
|  | Glucose (mg/DL) | 276.2 ± 17.40 | 309.2 ± 10.96 | 314.0 ± 10.66 ^∫^ | 255.8 ± 21.30 | 236.0 ± 21.60 |
| 8  w | TC (mg/DL) | 167.56 ± 13.78 | 256.37 ± 22.20 ^*,⧧,∫^ | 218.23 ± 24.89 ^∫^ | 189.76 ± 21.09 | 175.34 ± 24.19 |
|  | TG (mg/DL) | 77.75 ± 5.97 | 86.07 ± 8.29 | 79.36 ± 5.72 | 68.44 ± 6.25 | 84.82 ± 9.09 |
|  | HDL-C (mg/DL) | 53.38 ± 1.49 | 57.43 ± 8.21 | 56.42 ± 3.85 | 50.51 ± 1.91 | 49.16 ± 3.97 |
|  | LDL-C (mg/DL) | 98.63 ± 12.11 | 181.72 ± 13.93 ^*^ | 145.93 ± 22.53 | 125.57 ± 19.12 | 109.22 ± 21.58 |
|  | Glucose (mg/DL) | 261.4 ± 10.96 | 310.8 ± 15.89 ^∫^ | 317.2 ± 11.74 ^∫^ | 250.2 ± 15.18 | 211.2 ± 8.66 |

Values are means±SE, *p<.05; vs CO, ⧧p<.05; vs HFND, ∫p<.05; vs HFNDT-DT, TC; Total Cholesterol, TG; Triglyceride, HDL-C; High Density Lipoprotein-Cholesterol, LDL-C; Low Density Lipoprotein-Cholesterol, CO; Normal diet group, HF; High fat diet group, HFT-DT; High fat diet + Training + Detraining group HFND; Dietary change group, HFNDT-DT; Dietary change + Training + Detraining group.

Lipid profiles and glucose analysis

Plasma total cholesterol (TC) and triglyceride (TG) levels were analyzed with commercial TC and TG kits (Asan Pharmaceutical, Korea). High density lipoprotein cholesterol (HDL-c) level was analyzed with HDL-c kits (Shinyang Diagnostics, Korea) and Low density lipoprotein cholesterol (LDL-c) was calculated with the following equation: LDL-c = TC - (HDL-c + TG/5). Blood glucose level was estimated using a GlucoDr glucometer (Allmedicus, Korea).
