# Supplementary material for: Effects of detraining and retraining on muscle energy-sensing network and meteorin-like levels in obese mice
Source: Lipids Health Dis. 2018 Apr 27;17:97. doi: 10.1186/s12944-018-0751-3 (PMC5924483; doi:10.1186/s12944-018-0751-3)
Supplement: Supplementary file 3 — Table S3. Lipid profiles and glucose after 8 weeks of retraining. (DOCX 17 kb) [file 12944_2018_751_MOESM3_ESM.docx]

**Table S3** Lipid profiles and glucose after 4 weeks of retraining

|  | CO | HF | HFT-RT | HFND | HFNDT-RT |
| --- | --- | --- | --- | --- | --- |
| TC (mg/DL) | 152.76 ± 2.29 | 248.78 ± 22.37 ^*,⧧,∫^ | 246.73 ± 6.91 ^*,⧧,∫^ | 172.69 ± 9.98 | 127.53 ± 8.48 |
| TG (mg/DL) | 84.70 ± 13.63 | 108.91 ± 10.96 | 68.15 ± 4.69 ^∫^ | 115.98 ± 2.28 | 122.93 ± 25.88 |
| HDL-C (mg/DL) | 54.81 ± 3.60 | 65.12 ± 4.32 | 70.72 ± 7.92 | 55.83 ± 3.93 | 43.75 ± 2.65 ^#,ⵜ^ |
| LDL-C (mg/DL) | 81.01 ± 10.89 | 161.88 ± 25.67 ^*,⧧,∫^ | 162.38 ± 10.90 ^*,⧧,∫^ | 99.66 ± 18.96 | 59.19 ± 6.56 |
| Glucose (mg/DL) | 256.6 ± 11.21 | 328.2 ± 14.10 ^*,⧧,∫^ | 325.0 ± 12.32 ^*,⧧,∫^ | 252.0 ± 11.89 | 220.8 ± 7.88 |

Values are means±SE, *p<.05; vs CO, #p<.05; vs HF, ⵜp<.05; vs HFT-RT, ⧧p<.05; vs HFND, ∫p<.05; vs HFNDT-RT, TC; Total Cholesterol, TG; Triglyceride, HDL-C; High Density Lipoprotein-Cholesterol, LDL-C; Low Density Lipoprotein-Cholesterol, CO; Normal diet group (n=5), HF; High fat diet group, HFT-RT; High fat diet + Training + Detraining + Retraining group (n=5), HFND; Dietary change group (n=5), HFNDT-RT; Dietary change + Training + Detraining + Retraining group (n=5).

Lipid profiles and glucose analysis

Plasma total cholesterol (TC) and triglyceride (TG) levels were analyzed with commercial TC and TG kits (Asan Pharmaceutical, Korea). High density lipoprotein cholesterol (HDL-c) level was analyzed with HDL-c kits (Shinyang Diagnostics, Korea) and Low density lipoprotein cholesterol (LDL-c) was calculated with the following equation: LDL-c = TC - (HDL-c + TG/5). Blood glucose level was estimated using a GlucoDr glucometer (Allmedicus, Korea).
